# Design Rules of Bidirectional Smart Sensor Coating for Condition Monitoring of Bearings

**DOI:** 10.3390/polym15040826

**Published:** 2023-02-07

**Authors:** Van-Cuong Nguyen, Minh-Quyen Le, Sophie Bernadet, Yoann Hebrard, Jean-François Mogniotte, Jean-Fabien Capsal, Pierre-Jean Cottinet

**Affiliations:** 1 LGEF, INSA-Lyon, EA682, University Lyon, 69621 Villeurbanne, France; 2 Arc en Ciel Sérigraphie, Z.I Le Forestier, 42630 Regny, France; 3 SKF-Aerospace, 22 Rue Brillat Savarin, 26958 Valence, France; 4 Hybria Institute of Business and Technologies, Écully Campus, 69130 Écully, France

**Keywords:** multilayered smart coating, piezoelectric sensor, bidirectional load, screen printing, design rules, finite element simulation, condition monitoring

## Abstract

This paper reports a novel monitoring technique of bearings’ bidirectional load (axial and radial) based on a smart sensor coating, which is screen printed onto the surface of a cross-shaped steel substrate. To ensure the accuracy and stability of measurement as well as the durability of the printed coating, the developed prototype is built according to design rules commonly used in electronic circuits. The finite element model (FEM) is used to predict the mechanical property of the tested substrate under either unidirectional or bidirectional loads. Regarding the output voltage of the piezoelectric sensor, experimental results are revealed to be well-corelated to the numerical simulation. It is pointed out that the output signal generated from the sensor (electrode) could be particularly affected due to the capacitive parasite coming from the conductive tracks (CTs). Such a phenomenon might be reduced by printing them on the dielectric layer rather than on the piezocomposite layer. The study also investigates a highly anisotropic shape of electrodes (rectangular instead of circle), indicating that the orientation of such electrodes (axial or radial) does affect the output measurement. To sum up, the high performance of a sensor network coating depends not only on the ultimate characteristics of its own materials, but also on its structural design. Such an issue has been rarely reported on in the literature, but is nonetheless crucial to achieving reliable condition monitoring of bearings, especially for multidirectional loads—a key signature of early failure detection.

## 1. Introduction

Rolling element bearings are widely used to induce rotational motion, which is frequently a significant function of the overall system, such as wheels on a train or a rotor on a plane. The proper operation of a bearing over its designed life cycle is thus critical for ensuring product quality, preventing machine damage, and even preventing human death, especially in the field of aerospace [1,2,3]. Unfortunately, premature and unexpected bearing failures are common in real-world applications due to faulty installation, insufficient lubrication, overloading, and other unforeseeable adverse conditions [4]. For improved condition monitoring (CM) of bearings, various sensing and measurement technologies have been involved to provide operational indicators [5,6,7,8]. CM makes it possible to improve knowledge of the structures via better supervision, reducing interventions, and optimizing the materials used. The ultimate goal of CM involves the development of autonomous, continuous monitoring systems capable of detecting structure damage in real time to avoid any accidents. The key challenges are in the early detection of damage, allowing optimal maintenance and, as a result, reduced cost. There are various sensing methods to detect faults in the bearings, such as vibration monitoring [9,10], wear debris monitoring [11], temperature monitoring [12,13], non-destructive testing [14,15], and so on [16,17]. However, some methods are not effective for determining the early faults’ condition. For instance, vibration sensors are the most common technique used for the CM of bearings, but they only provide indirect indicators that need to be post-processed to convert them into useful representations [18]. Similarly, acoustic emission sensors—typically used to perform an analysis of load torque, rotational speed signal, magnetic field frequency, etc.—could allow for the detection of a two-dimensional (2D) signal system. Nonetheless, complex mathematical calculations, a numerical model, or even a neural network would need to be carried out to transform signals detected by the sensors into 2D images that could then be analyzed and diagnosed [19]. In order to enhance productivity and reduce maintenance cost, an online monitoring system is essential to check the status of bearings.

This paper proposes an alternative method to detect online and predict the early failure of bearings via direct indicators by monitoring bidirectional (2D) load thanks to a smart sensor coating. Choosing load as an indicator is relevant, as any change in bearing condition (structural damage) directly affects the load signal [20]. Among various smart sensor types, piezoelectric materials have been chosen because of their ability to respond to any stiffness change [21,22,23]. Actually, such materials have been intensively exploited in various industrial fields [24]. For a smart coating, a composite of barium titanate (BaTiO_3_) nano-powder is chosen because of its lead-free property, high permittivity, easy process, and effective cost [25,26]. The composite consists of BaTiO_3_ fillers randomly dispersed within Polyurethane Acrylate (PUA) thermoplastic, i.e., considered as one of the most popular curable resins. Besides requirements in the selection of piezoelectric materials, a geometric constraint imposed upon sensor circuit design is also essential to ensure it functions properly, reliably, and can be produced with an acceptable yield. One of the main objectives of this work is dedicated to the basic design rules applied on the sensor circuit. Eventually, this analysis allows the prevention of undesired effects that could dramatically trouble the output measurement, or even the electrical breakdown of the device when being subjected to a high poling voltage. Accordingly, design rule checking seems to be an important stage to ensure good functionality of the sensor, but nonetheless has been rarely reported in the literature.

To achieve the full performance of bearings’ CM, the developed sensor must fulfil the following criteria: mechanically flexible, adaptable to different sizes and shapes, reasonable cost, easy process, and low environmental impact. In this situation, the printing technique becomes a viable option for achieving the deposition of multiple-layered materials in additive manufacturing (AM) that are of various sizes and shapes [27]. In particular, among the various methods currently in use [28,29,30,31,32], screen printing appears to be a quick, effective, straightforward, durable, and inexpensive process that may be used on an industrial scale [33,34,35]. By using screen printing, the polymer-based piezoelectric composite is directly screen printed onto the surface of the host structure, which is assimilated to the steel outer ring of the bearing. Because of electromechanical coupling properties, a voltage signal can be generated through the piezoelectric sensor when being subjected to a mechanical strain [36]. The mechanical properties of the entire system are mostly determined by the characteristics of the steel-type material because the typical sensor layer is very thin compared to the underlying substrate. In our previous work [37], we investigated a four-point bending (4 PB) setup [38,39] that allowed us to generate consistent and uniaxial stress along the longitudinal direction of the instrumented substrate. Another advantage of this technique is the ability to directly determine the transverse piezoelectric constant (*d*_31_), which is usually found through the intermediary longitudinal constant (*d*_33_).

In this study, we extend the 4PB approach to monitor bidirectional load (i.e., axial and radial). As a result, the sensors need to be mounted on a specific structure capable of detecting any mechanical change along two orthogonal axes [40]. A solution relying upon the use of a cross-shaped subtract is investigated to testify the sensor ability in response to the bidirectional stress. A finite element model (FEM) is developed using ANSYS software to figure out the mechanical behavior of the cross steel substrate in different configurations (unidirectional or bidirectional with an asymmetric/asymmetric feature). Due to the typically thin sensing coating in comparison to the underlying substrate, the mechanical properties of the whole system are mainly determined by the characteristics of the steel-type material. Therefore, the effective piezoelectric behavior of the thin composite could be reliably inferred by using a 4PB setup. Practical tests allowed us to assess the sensor performance, which might be affected by several factors comprising length of conductive tracks and shape of electrodes. Understanding the role of each factor is of outstanding contribution to improve the structural sensing design so as to match specific requirements of bearings’ CM.

## 2. Process and Architecture of Bidirectional Sensor

### 2.1. Concept of Smart Bearing

In the aeronautic field, bearings are among the most stressed components of aircrafts and are a frequent source of failure. Providing the best availability of aircrafts is a key driver in the aeronautics industry. A monitoring system able to detect signs of failure before they happen, thanks to sensors and diagnosis/prognosis algorithms, is of primary significance for improving aircraft operability [41]. The usual failures that can be encountered are improper lubrication, corrosion, contamination, etc. In most cases, bearing defects are manifested by a more or less material removal on a mat surface such as an inner/outer ring, or even rolling elements [4,42]. They generally lead to several mechanical effects in machines, especially an increase in the load applied to the rolling elements. It is thus necessary to monitor not only the magnitude of the load but also its directions, for easier maintenance [43]. *Radial load* (also known as transverse force) is perpendicular to the shaft’s longitudinal axis, whereas *axial load* (or thrust load) is parallel to this axis. Some bearings can withstand a combination of radial and axial loads applied to the shaft, to some extent [43]. Usually, in a bearing system, the rolling element is subjected to periodic deformation (local deformation) occurring on the outer ring, in addition to the deformation due to the external force. Thus, during the rotational motion of bearings, maximum deformation occurs when the rolling element is co-axial (in phase) with the sensor (measurement point of charge displacement), while minimum deformation occurs when they are out of phase.

**Figure 1:**
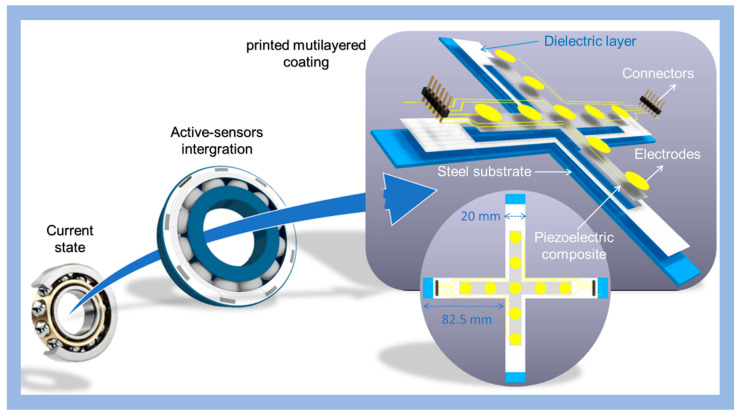
From the current state of bearings to a new generation coated with bidirectional printed sensor for condition monitoring.

As illustrated in Figure 1, a cross-shaped substrate coated with a piezoelectric sensor was developed in this study to examine the performance of loading in two directions (axial/radial) on the characteristics of the tested sample. The steel substrate, provided by SKF-Aerospace, was used to simulate the mechanical property of a bearing. Three thin layers were printed on the cross substrate, such as dielectric layer, piezoelectric sensor, and conductive tracks (CTs), together with electrodes. The electrodes of 10 µm thickness, designed with a circular (12 mm diameter) or rectangular (12×3 mm^2^) shape, were deposited on the piezoelectric layer. The CTs (with 10 µm thickness and 0.7 mm width) were printed on the dielectric layer (DL) to prevent any measure perturbation caused by their nonnegligible length. The influence of the CTs, as well as the efficiency of the DL, will be discussed in Section 4.2. As seen in Figure 1, the piezoelectric layer covers most of the surface of the outer ring, whose deformation induced by load is monitored via the surrounded electrodes linking to an electrical connector. For the sake of simplicity, characterization tests were performed on the piezoelectric layer coated on the flat crossed-shape substrate. Such an architecture allowed for an assessment of the applied load along radial and axial directions, which are supposed to be similar to what would happen on the bearing surface.

### 2.2. Rules of Sensor Network Design

**Figure 2:**
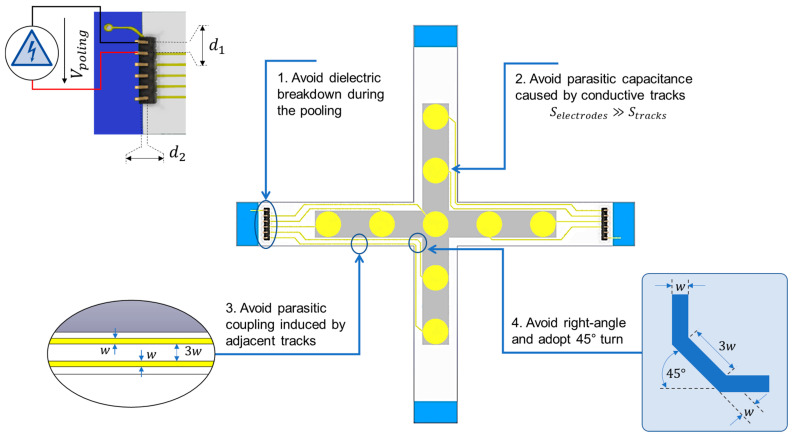
Four principal rules of sensor network design.

The whole system design acts as an electrical network where a change in electric field could be critical for the sensor’s life and performance. Accordingly, to make the sensor device work correctly, the printed components must meet requirements of basic electronic design rules. The four principal rules, as summarized in Figure 2, set specific certain geometric and connectivity restrictions to warrant sufficient margins to account for variability in the AM process. The description of these four rules is outlined as follows:

(1) *Could the applied voltage during polarization cause any arcing in the air?* The first rule, given to avoid dielectric breakdowns during polarization, concerns the space of the pin connector (namely *spacing rule*). In our case, the sample is polarized under a poling field of $E_{p}$ = 6 V/µm (at 80 °C for 20 min). Considering a 20 µm coating thickness, the applied bias voltage is thus equal to $V_{p}$ = 120 V. The permittivity of an ideal dielectric (e.g., air) is revealed to be constant; nonetheless, with a strong-enough electric field, all practical dielectrics fail in this respect. The failure is typically abrupt and is noticed as a sharp rise in conductivity, indicating that electrons are successfully ejected from their host molecules. The threshold value of the electric field intensity at which this occurs is known as dielectric strength, and the sudden change in behavior observed with an electric field greater than the threshold value is known as a dielectric breakdown. Typical dielectric breakdown of the air is considered to be 3 V/µm. Such a critical value should be avoided at all costs by verifying whether or not the distance between two adjacent pins $\left(\mathrm{denoted}\;d_{1}\right)$, and the one between the pin and ground substrate $(\mathrm{denoted}\;d_{2}),$ is not too small to provoke any breakdown. As the chosen connector has a pitch of 2.54 mm and an enclosure of 0.8 mm (typical parameters of commercialized available connector), the corresponding electric fields are calculated as:

(1) $$d_{1}=2.54\;\mathrm{mm}\;\to \;E_{1}=\frac{V_{p}}{d_{1}}\approx 0.05\;V/µm\;\;d_{2}=0.8\;\mathrm{mm}\;\to \;E_{2}=\frac{V_{p}}{d_{2}}\approx 0.15\;V/µm$$

These values are obviously far from the critical breakdown occurring in the air (3 V/µm), so the chosen connector entirely meets the requirement of the *spacing rule*.

(2) *Does the capacitive effect induced by conductive tracks (CTs) affect the measurement?* To prevent parasitic capacities that could disturb measurement, the second rule focuses on the relationship between the surface of electrodes and the one of CTs [44] (namely *surface rule*). It is well-known that parasitic capacitance is usually unavoidable, existing between the parts of the electronic component or circuit simply due to their proximity. When two different voltage electrical conductors (such as CTs) are close together, the electric field between them somehow causes a stored electric charge, which is the main origin of parasitic capacitance. To reduce its impact, it is necessary to ensure that the surface of the sensor (i.e., electrode, $\mathrm{denoted}\;S_{e}$) is much larger than that of the CTs ($\mathrm{denoted}\;{\;S}_{t}$). The ratio $\left(r_{C}\right)$ between the sensor’s capacitance ($\mathrm{denoted}\;C_{e}$) and the track’s capacitance ($\mathrm{denoted}\;C_{t}$) can be expressed as:

(2) $$r_{C}=\frac{C_{e}}{\;C_{t}}=\frac{\varepsilon_{composite}\;}{\varepsilon_{dielectric}}\times \frac{S_{e}}{{\;S}_{t}}\times \frac{d_{t}}{d_{e}}$$

where $d_{t}\;\left(\sim 10\;µm\right)$ and $d_{e}\;\left(\sim \;20\;µm\right)$ are the thickness of the track and of the electrode, respectively. Even though the permittivity of the dielectric layer ($\mathrm{denoted}\;\varepsilon_{dielectric})$ is much smaller than that of the composite $\left(\mathrm{denoted}\;\varepsilon_{composite}\right)$ [45], to ensure that $C_{sensor}\gg C_{track}$, each electrode must have a significant area as opposed to its own track liking to the connector. In practice, $r_{C}$ is chosen to be greater than 10 to minimize the parasite effect. Anyway, the *surface rule* must be fulfilled, even in the case of electrodes associated with the longest track.

(3) *How to avoid parasitic coupling induced by adjacent tracks?* The third rule is dedicated to the separation between two adjacent tracks with respect to their own width (*spacing rule*). When two electrical circuits are in the vicinity of one another, a signal propagating in one circuit can induce a signal in another circuit, due to capacitive (electric field) and/or inductive (magnetic field) coupling between them. This phenomenon is referred to as crosstalk. The general rule says that the separation between the tracks should be at least 3w, where *w* is the width of the track (Figure 2). This practice helps to reduce crosstalk and coupling between adjacent tracks on the same layer [46,47]. For a specific application relating to the instrumentation of the bearing system, compactness is one of the primary criteria. Respecting *spacing rule* is a true challenge, especially when the number of sensors and tracks is important. A compromise must be taken into consideration for achieving properly reliable measurement as well as high sensing performance.

(4) *How to design the track’s corner?* The last rule concerns the critical position, where CT encounters an important bend (namely the *angle rule*). While single discontinuities such as sharp corners make little difference, cumulatively, they have a significant impact. A sharp track corner creates shunt capacitance to the ground plane, which degrades insertion loss and increases the capacitance. This phenomenon results in a change in characteristic impedance that involves reflections of signal, particularly in a high-frequency domain [48,49]. Therefore, track corners should be as round as possible with a radius no tighter than the differential pair separation. If smooth curves are not possible at the corners, a design with cumulative turns of 45° could be acceptable.

### 2.3. Material Selection for Piezoelectric and Conductive Inks

Electronic circuits require very specific functional characteristics, and this continues to be a challenge for printing techniques. In order to overcome this challenge, two different approaches can be distinguished: process engineering, where the currently existing materials are tailored in terms of shape, geometry, and interconnectivity [50,51,52,53,54,55], or material engineering, where new materials with tailored functional characteristics for each application are developed [56,57,58]. In particular, for ferroelectric materials, the main approach for tailoring electromechanical coupling is to include bulk fillers with large piezoelectric constants into the polymeric matrix [59,60,61]. Polymers offer advantages such as easy processability or good mechanical properties, while ceramic filler provides high piezoelectric features. The most used ferroelectric filler for this purpose is barium titanate, BaTiO_3_, crystallizing in a perovskite structure, as it is a lead-free ceramic with a high dielectric and piezoelectric constant, depending on its purity, grain size, temperature or preparation method [62]. In the case of polymers, polymethylmethacrylate [63], polyetheretherketone (PEEK) [64], polystyrene [65], and polyvinylidene fluoride (PVDF) [66] have been used for the development of those composites.

In the context of materials for printed electronics, photopolymerizable and UV-curable polymers emerged as good alternatives to solvent or melting processes due to the advantages of the photopolymerization process, including room curing temperature, curing times in minutes or seconds, reduced VOC (volatile organic compound) emissions and space and energy efficiency, among others [67]. To develop the piezoelectric ink, Polyurethane Acrylate (PUA), one of the most popular photocurable resins based on the thermoplastic PU, was chosen as the polymer matrix. PUA has attracted much attention in ultraviolet (UV) curable coatings attributed to its excellent flexibility, prominent adhesion on substrates, and a variety of adjustable features, ensuring adhesion on the metal surface of the bearing [37,68]. Significant results can be acquired when it is applied in coatings for metals or other electronic products [69]. Photopolymerization and UV curing represent fast and solvent-free techniques to obtain polymer-based composites, being two of the effective methods used in the polymerization process. Up to now, just a few studies on UV-curable dielectric materials have been reported, mainly focusing on their electrical properties [70,71]. Later, Mendes-Felipe et al. provided further characterization on the morphological, thermal, mechanical, and dielectric properties of BaTiO_3_/PUA composites as a function of the particle content and size [72]. However, none of them investigated the piezoelectric properties of such materials, which are somehow of high interest in various sensing device applications.

In this study, the piezoelectric ink was developed according to a collaboration between LGEF and VFP ink technologies. The composite consisting of BaTiO_3_ nano-powder, randomly dispersed within the PUA matrix with a concentration of approximately 20 vol%, is suitable for additive manufacturing (AM) processes thanks to a light-controlled polymerization system [73]. The electrodes and conductive tracks (CTs) are made of silver ink (LOCTITE ECI 1010 E&C, commercialized by Henkel Technology, France), with good adhesion, high electrical conductivity, and viscosity of approximately 9 Pa.s, adaptable to screen printing technology.

### 2.4. Printing Process

**Figure 3:**
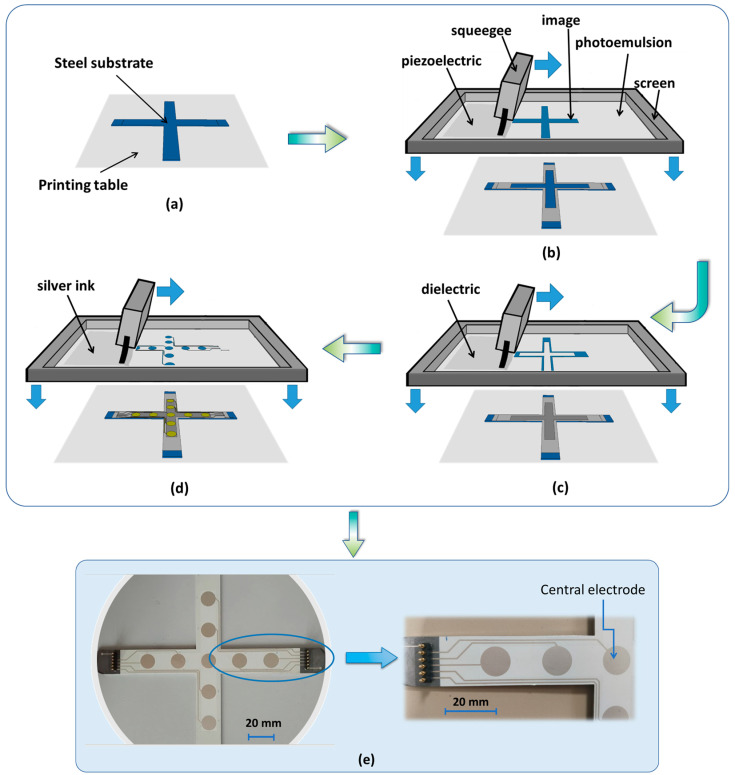
Fabrication of two-axis piezoelectric sensor via screen printing process: (**a**) setting steel substrate; (**b**) printing piezoelectric layer; (**c**) printing dielectric layer; (**d**) printing electrode and conductive tracks; and (**e**) full printed coating with electrical connectors.

Figure 3a–d illustrates the fabrication of the multilayered piezoelectric sensor coated on a cross-shaped steel substrate. The three layers were screen printed via an industrial process piloted by ACE (Arc en Ciel Sérigraphie). The printing machine is a ¾ automatic ATMA AT600H/E composed of a mesh screen, inks, and a squeegee to transfer a stenciled design onto a flat fabric surface. The printed layer thickness, as well as the pattern definition, depended on the fabric nature and the mesh. Regarding the prototype design, the printing frame was chosen with 230 meshes and the fabric was made of polyester. Corresponding to each layer, a suitable ink was used, comprising PUA polymer ink (used for the dielectric layer), BaTiO_3_/PUA composite ink (used for the piezoelectric layer), and silver ink (used for the electrode and conductive tracks). Each layer required a specific curing process. The best electrical properties of the electrode were met by performing thermal curing of the conductive silver ink in a drying oven (SIEBDRUCK TRO II) between 100 and 150 °C. For the dielectric layer, the excellent insulating characteristics required UV curing at a power of 500 mJ/cm^2^, carried out in a tunnel (SILAIR). To achieve optimal properties such as resin curing, the piezoelectric layer must be also exposed to a UV light (405 nm wavelength), with lower power of 300 mJ/cm^2^ of irradiance at room temperature. The proposed printing technique allows to create a very thin film sensor, at the order of 20 µm, leading to an easier polarization process and reduced breakdown probability [74,75].

The final customized product is shown in Figure 3e, where a 6-pin electrical connector is implemented on the extremity of the dielectric layer, linking to the conductive tracks. The distance between two successive pins is large enough (~2.54 mm) to avoid any electrical arc in the case of a high voltage application.

## 3. Simulation and Experimental Setups

### 3.1. Polarization Setup

Before performing the empirical characterization, the coating sensor needs to be polarized to create a formation of the dipoles, in which their remanent polarization is kept even when the voltage is OFF. This effect is a responsibility of piezoelectricity, i.e., a macroscopic phenomenon relating to the intrinsic dipole’s orientation of particles. An increase in the molecular mobility of the amorph phase of the matrix, i.e., above the glass transition temperature, allows the orientation of the electrical dipoles of the particles along the poling field. In our case, the polarization was performed in the air with an optimal condition (i.e., poling field of 6 V/µm at 80 °C for 20 min) that has been thoroughly reported on in our previous work [37].

The poling setup consisted of a waveform generator (Agilent 33210A, Keysight Technologies Inc.) coupled with an amplifier (10/10 B-HS, TREK Inc., Medina, NY, USA) by a factor of 1000. The DC electric field (*E*) was progressively increased until the desired value was met and was kept constant for a few minutes. For the polarization at a high temperature, the entire sample holder was placed into an oven (Votsch Industrietechnik TM, VT7004), which allowed for the control of temperature with high precision. Afterwards, samples were progressively cooled down at room temperature under the field. As soon as the field was removed, samples were short-circuited for 5 min to totally evacuate the undesired electrostatic charge. To check the success of the polarization procedure, the piezoelectric sensitivity (*d*_33_, usually defined as the longitudinal charge coefficient) was measured using a piezometer (Ye2730a–D_33_ Meter).

### 3.2. Four-Point Bending (4PB) Setup

**Figure 4:**
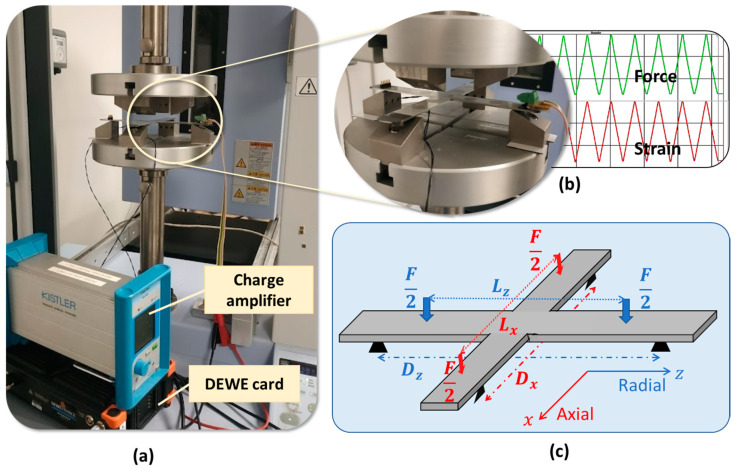
Setup of four-point bending (4PB) test using Shimadzu press: (**a**) measurement bench; (**b**) zoom-in on sample’s implementation; and (**c**) test could be performed by adjusting force and distance parameters in *x*- and *z*-axis.

To facilitate the characterization, a four-point bending (4PB) apparatus was employed in practice to produce bidirectional and uniform stress on the tested substrate along the *x*- and *z*-axis. Regarding the very thin layer of the printed coating (about 20 µm) with its excellent adhesion to the substrate surface, the deformation of the coating layer in both directions could be supposed to be similar to the substrate’s one. As shown in Figure 4a, the 4PB tests were conducted, under room temperature, on a full specimen (substrate/sensor) using a SHIMADZU press (AGS-X). The biaxial cross sample, being held by four supports underneath, was subjected to four identical forces perpendicular to the tested substrate (see Figure 4b).

The desired DC or AC load vertically applied to the bending fixture was quantified via a load sensor (HBM, 10 N–10 kN). The central electrode (seeFigure 3e), mostly affected by both directions, was chosen for test measurements. The other electrodes would have higher responses in their preferred direction, which is somehow similar to the case of the uniaxial sensor investigated in previous work [37]. As a result, studying the central electrode allows one to better highlight the bidirectional effect of the cross-sensor coating. Two strain gages (RS PRO) were thus coated on the middle bottom of the specimen, in such a way that they allowed one to determine the deformation in both radial (*z*-axis) and axial (*x*-axis) directions (cf. Figure 4c). Such a deformation does not only depend on the applied force, but also on parameters of distance: $D_{x}$ and $D_{z}$ are the supports’ span while $L_{x}$ and $L_{z}$ are the span between the two applied forces, corresponding to the *x*- and *z*-axis. The mechanical characteristics of the substrate could be therefore deduced in different configurations. In addition, the charge output delivered from the piezoelectric sensor was monitored via a charge amplifier (KISTLER, Type 5015). Finally, real-time signals were simultaneously acquired and recorded through a Sirius 8XSGT card interfaced with the DEWE software. Post-data treatment was performed with MATLAB and Excel.

### 3.3. Simulation Implementation

**Figure 5:**
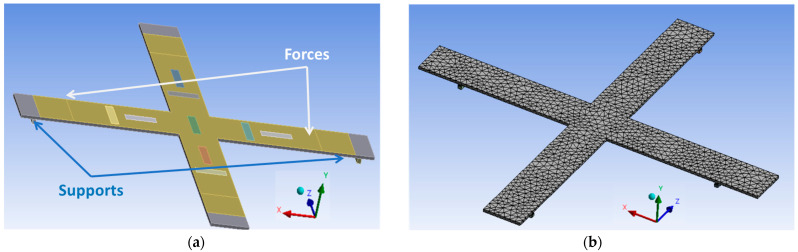
FEM of the bidirectional coating-sensor-based COMSOL multiphysics: (**a**) 4PB configuration; (**b**) mesh pattern for physical simulation.

Figure 5a illustrates the 3D geometry model of the tested cross sample. In either the *x*- or *z*-axis, each beam comprises four main components including bottom support rollers, the steel substrate (beam), the piezoelectric layer with five electrodes (circle or rectangular shape), and top blades applied by the external load. The support spans ($D_{x}$ and $D_{z}$) together with the load distances ($L_{x}$ or $L_{z}$) can be varied. A 4PB model was built with the same dimensions as the real sample. The properties of the entire specimen used in the finite element model (FEM)-based ANSYS software are shown in Table 1. All piezoelectric composite parameters were determined using empirical measurements. The piezoelectric constants (*e*_31_, *e*_33_, and *e*_15_) along either the *x*- or *z*-axis were computed as a function of Young’s modulus (*Y*) and other piezoelectric constants (*d*_33_ and *d*_31_):

(3) $$\left\{\begin{array}{c} e_{31}=d_{31}\left(Y_{11}^{E}+Y_{12}^{E}\right)+d_{33}Y_{13}^{E}\\ e_{33}=2d_{31}Y_{13}^{E}+d_{33}Y_{33}^{E}\\ e_{15}=d_{15}Y_{44}^{E} \end{array}\right.$$

Note that the piezoelectric constants *e* and *d* represent the sensitivity of the charge detection in response to a mechanical strain and stress, respectively. As the BaTiO_3_ fillers are randomly dispersed within the PUA matrix, the composite was supposed to be isotropic, meaning that its properties are directionally independent. Considering no shear load is applied to the specimen (i.e., $d_{15}=0$), Equation (3) can thus be simplified as:

(4) $$e_{15}=0,\;\;\;\;\;e_{31}=e_{33}=d_{33}Y\left(2\nu +1\right)$$

The above equation allows for the estimation of the value of the piezoelectric constant implemented on the FEM (see Table 1). The bottom of the substrate is electrically grounded. The purpose here is to determine, for each distance of loads and supports, the mechanical strain as well as the potential change induced on the electrodes when the specimen is subjected to external loading.

As shown in Figure 5b, the mesh built for the cross model was triangular in shape and heterogeneous, with a finer mesh at the sensor–substrate interface where the highest electrical gradients were expected. To obtain the high discretization quality of the modeled system, mesh should be as fine as possible [76,77]. The problems encountered when refining the mesh were mainly related to the connection of the structure, but also to the limits imposed by the software or by the computer. As a result, a compromise between the accuracy of the numerical resolution and the calculation time must be considered.

## 4. Results and Discussions

### 4.1. Simulation Results

**Figure 6:**
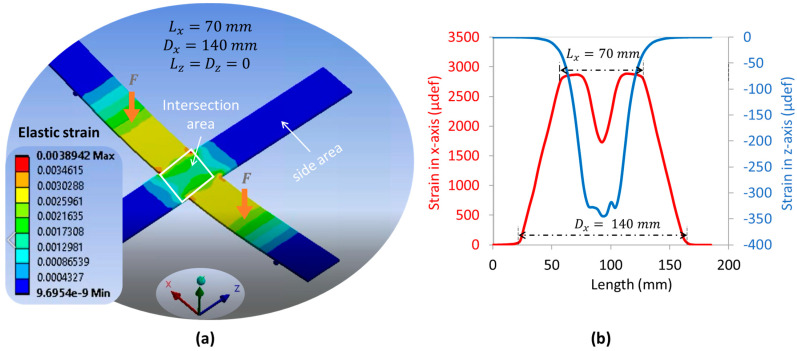
Unidirectional model using FEM: (**a**) four-point flexure design with load applied on *x*-axis only; (**b**) strain in both x- and *z*-axis versus their own length.

**Figure 7:**
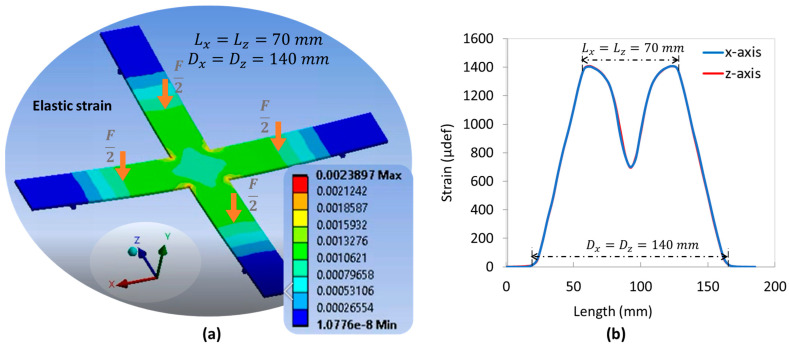
Symmetric bidirectional symmetrical model using FEM: (**a**) four-point flexure design with load applied on x- and *z*-axis ($L_{x}=L_{z}$ and $D_{x}=D_{z}$); (**b**) strain in both *x*-axis and *z*-axis versus their own length.

**Figure 8:**
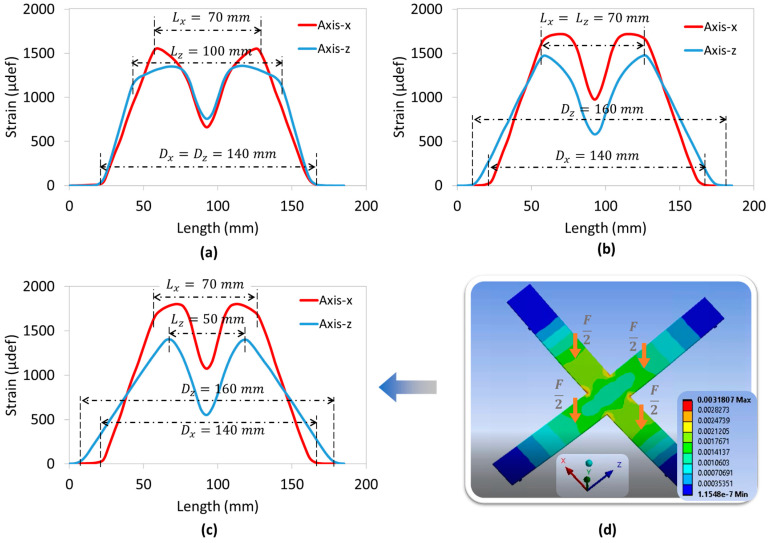
Asymmetric bidirectional model using FEM: strain in both *x*-axis and *z*-axis versus their own length with (**a**) $L_{x}\neq L_{z}$ and $D_{x}=D_{z}$; (**b**) $L_{x}=L_{z}$ and $D_{x}\neq D_{z}$; and (**c**) $L_{x}\neq \;L_{z}$ and $D_{x}\neq D_{z}$, where numerical image is shown in (**d**).

To assess the mechanical strain of the cross substrate in axial (*x*-axis) and radial (*z*-axis) directions, the 4PB method based on FEM was investigated. All tests were performed under a constant total load of 250 N (i.e., denoted as 2F), which was equally divided for application to the four arms. By tuning the distance parameters between the supports ($D_{x}\;\mathrm{and}\;D_{z}$) as well as the applied load ($L_{x}\;\mathrm{and}\;L_{z}$), it was possible to distinguish between the three following configurations:

- Unidirectional load (Figure 6a): the mechanical load is applied in one direction only (e.g., axial) where $L_{z}=D_{z}=0$, meaning that neither support nor load is involved in the z-direction.
- Symmetric bidirectional load (Figure 7a): the mechanical load is uniformly applied in both radial (*z*-axis) and axial (*x*-axis) directions where $L_{x}=L_{z}$ and $D_{x}=D_{z}$.
- Asymmetric bidirectional load (Figure 8d): The mechanical load is applied in the axial and radial directions but is not uniform ($L_{x}\neq L_{z}$ and/or $D_{x}\neq D_{z}$). To better compare and assess the influence of each parameter, three settings are shown in Figure 8a–c.

For the sake of simplicity, the support span is set to be superior to the load span, meaning that $D_{x}>L_{x}$ and $D_{z}>L_{z}$. Inverting the sense of these inequalities makes the strain invert its sign, but not its magnitude. The evolution of the mechanical strains along the two axes (denoted $S_{x}\;\mathrm{and}\;S_{z}$) is illustrated in Figure 6b, Figure 7b and Figure 8a–c, i.e., correspondingly for each configuration. The pertinent concluding remarks are summarized in Table 2.

**Figure 9:**
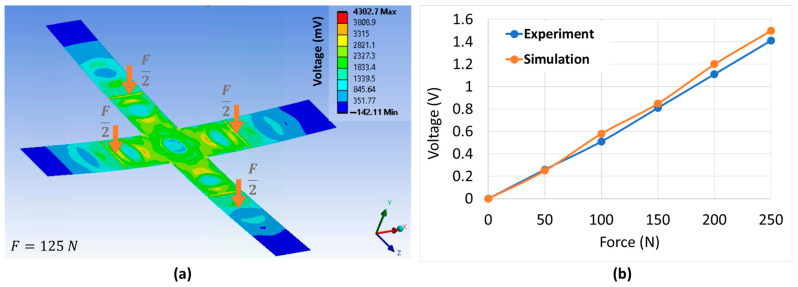
Comparison of the output voltage between experiment and simulation.

To assess the piezoelectric performance, a total upward load from 0 N to 250 N (with a step increment of 50 N) was applied to the cross substrate, and the generated output voltage at the central electrode was recorded (Figure 9a). A similar practical test was conducted, allowing one to compare the voltage values measured from the bending test and those obtained by the simulation. As confirmed in Figure 9b, the experimental potential output of the piezoelectric sensor shows very good linearity with the loading level, that well corelates with the numerical output predictions of the piezoelectric materials [37]. Under 250 N, the measured potential of the sensor is found to be approximately 1.4 V, while the corresponding simulation is 1.5 V. The experimental outputs are somewhat smaller than the model estimation, and such a slight discrepancy may come from several factors such as material properties, measurement setup, and force application method.

### 4.2. Experimental Results

#### 4.2.1. Linearity of Piezoelectric Response

This study aims to assess the linearity of the piezoelectric behavior in response to a given mechanical stress, which strongly depends on the three configurations previously introduced in Section 4.1: unidirectional load, and symmetric and asymmetric bidirectional load.

**Figure 10:**
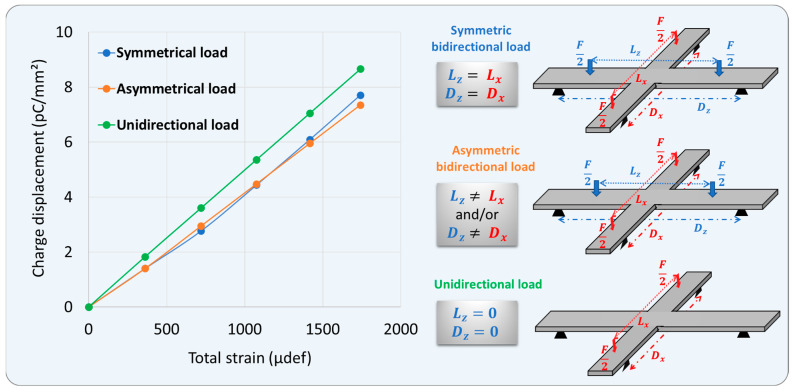
Charge displacement measured at the central electrode as a function of its total strain under three configurations of 4PB: unidirectional load, and bidirectional load with symmetry or asymmetry.

Figure 10 depicts the piezoelectric charge displacement of the central electrode as a function of the total strain, which is the sum of the axial and radial strain values. The input load is controlled by a triangular and periodic waveform whose amplitude varied from 0 to 250 N, with an increment of 50 N. For all configurations, excellent linearity of the piezoelectric response was achieved, reflecting the high sensing performance of the printed coating in a detection of either unidirectional or bidirectional load. Interestingly, the uni-axis gives rise to higher total strain than the multi-axis, resulting in a little higher charge displacement. This observation somehow corelates to the simulation reported above where the strain of the center is revealed to be drastically dropped in both directions. Such an effect, nonetheless, does not much affect the piezoelectric charge coefficient estimated from the slope of the characteristics curve as shown in Figure 10. Concerning the bidirectional load, both symmetric and asymmetric setting parameters lead to the same measures in the total strain and the charge displacement. Finally, all of these results confirm the high robustness of the 4PB method as well as the stable piezoelectric response of the sensor coating prototype, whatever the level and the direction of the input load.

#### 4.2.2. Effect of Conductive Tracks

**Figure 11:**
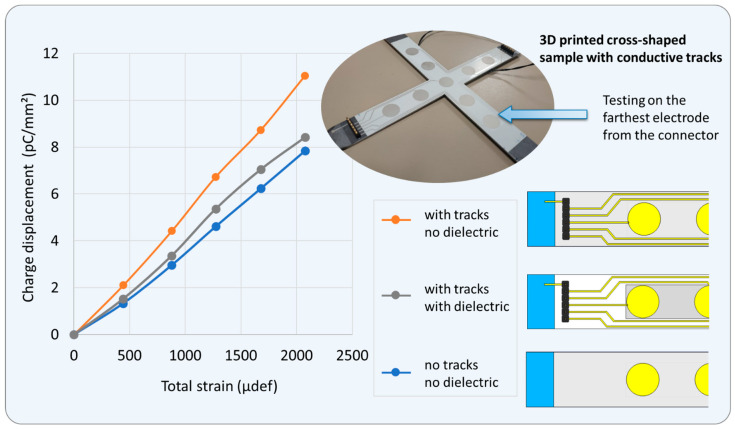
Piezoelectric response of three tested samples with or without conductive tracks (CTs) and dielectric layer. Measurements were performed on peripherical electrode that has the longest CT.

With the aim of evaluating the effect of the conductive tracks (CTs) on the piezoelectric sensing performance, test measurements were performed on the side electrode that led to the farthest distance from the electrical connector (see Figure 11). For other electrodes with shorter CTs, the effect of CTs would be smaller, but an efficient treatment to eliminate the impact of these tracks was still considered to ensure the correct signals of the sensors. As previously stated in Section 2.1, a simple solution based on the integration of a dielectric layer (DL) became involved. For a better comparison, three designs were performed as described in Figure 11:

- No tracks no dielectric (namely NTND): Only circle electrodes are stacked on the piezoelectric layer; no conductive track or dielectric layer is needed. This design is considered as the simplest one that leads to the best accuracy in empirical measurement. This is why it is preferred to be employed in lab-scale characterizations. On the industrial scale, however, CTs are needed to perform online monitoring via the sensing device printed on the bearing surface. After being implemented, the sensor coating might not be dismantled out of the bearing system.
- With tracks with dielectric (namely WTWD): CTs are coated on the DL to minimize their effect with respect to the active area defined by the electrodes, which are in turn coated on the piezoelectric layer. This architecture is built in a full configuration composed of four layers, which is intentionally designed to be integrated in the bearing system (see Figure 1).
- With tracks no dielectric (namely WTND): A sample with CTs and electrodes all printed on the piezoelectric layer, as no dielectric layer is implemented. This design allows one to find out whether or not the CTs have an impact on the sensor’s output signal.

Regardless of the load level (varying from 0 to 250 N), all samples resulted in the same total strain, confirming that both CT and DL do not much affect the mechanical properties of the sensor coating. Interestingly, the two samples with CTs have a higher charge displacement than the NTND sample (blue curve), demonstrating that CTs do disturb the electrical measure of the sensor. Although the diameter of the tracks is revealed to be small enough (0.7 mm width), their length is nonetheless considerable, particularly when they pass over the most deformed regions of the sample. As it can be seen, the WTND sample gives rise to an increase of 40% in the piezoelectric sensitivity (i.e., reflected by the slope of the orange curve displayed in Figure 11) as opposed to the NTND counterpart (blue curve). Figure 11 indicates a great contribution of the dielectric layer (DL) in correcting the sensor output signal troubled by the significant-length tracks. Indeed, a presence of DL in the WTWD design (gray curve) has successfully compensated the CT impact. A discrepancy of only 9% was observed with respect to the referenced sample without tracks (NTND), contrarily to 40% as in the case of without the dielectric layer (WTND). Actually, the fact of introducing the DL did not allow us to totally overcome the CTs’ impact on the charge measurement, which might be a result of small dielectric losses in the DL itself. However, regarding the measured uncertainties (of around 10%), a 9% discrepancy between the WTWD and NTND samples seems to be a satisfying result, confirming that the implementation of DL is of primary importance in a real device.

#### 4.2.3. Effect of Electrode Shape

**Figure 12:**
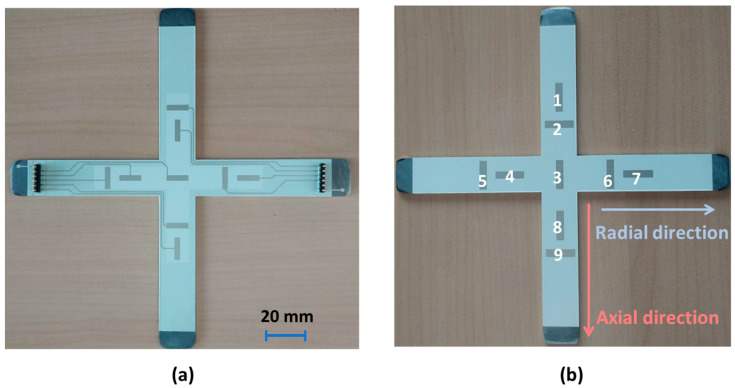
Cross-shaped sensor network coated with rectangular electrodes: (**a**) full printed coating with CTs and DL; (**b**) simple coating without CTs and DL.

The previous experiments were carried out on samples printed with circle electrodes whose shape was revealed to be directionally independent. In other words, the mechanical strain measurement of these circle sensors is similar in both axial and radial directions, and so is the charge displacement. To highlight the effect of the sensor’s shape on the output signal, electrodes were designed as an anisotropic rectangular shape (12×3 mm^2^) with a sufficiently high aspect ratio (factor four between the two sides). A full printed coating composed of nine rectangular sensors is displayed in Figure 12a, where the sensors’ length can be put along either the axial or radial direction. With the aim of separating the effect of the conductive tracks (CTs), practical tests were performed on the simplest design with neither CT nor electrical contact. Consequently, the out signals of the sensors were acquired through two “permanent magnet” connectors attached on the electrode and the steel substrate, which are conductive enough to be considered as electric ground. The selected magnets are revealed to be good conductors, and ensure excellent continuity of the whole circuit.

**Figure 13:**
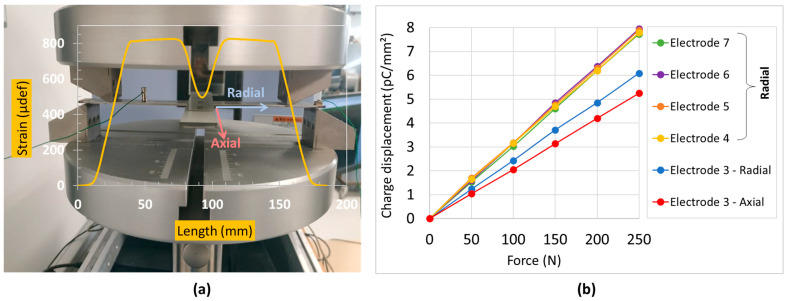
Four-point bending unidirectional test of cross substrate coated with rectangular sensors: (**a**) mechanical strain measured at the central electrode; (**b**) evolution of the charge displacement in response to the mechanical load applied to different electrodes. For the central electrode, the measurement was carried out on both axial and radial directions.

The electrodes are numbered from one to nine as depicted in Figure 12b. For the sake of simplicity, the experiment was only set in the unidirectional configuration, e.g., in the radial axis as described in Figure 13a. It can be observed that the induced strain (computed from the numerical model) drastically drops at the center and seems to be constant at the two sides. This explains why the piezoelectric response of Electrode 3 (blue curve) is lower than the one acquired from the “side electrodes” along the radial direction (i.e., Electrodes 4, 5, 6, and 7, shown in Figure 13b). Interestingly, for a given applied load, these electrodes attain identical output electrical charge (and thus the piezoelectric sensitivity), even though they are not oriented in a similar direction. Concretely, Electrodes 5 and 6 are placed along the axial direction while Electrodes 4 and 7 are placed in the radial one. As a consequence, the orientation (and thus the shape) of the sensor does not influence the measurement, if it is located in the area induced by constant deformation. Inversely, for Sensor 3, whose strain strongly varies at the electrode’s surface, its electrical signal might be affected by its orientation and shape. It is therefore interesting to perform the response of Sensor 3 in the axial direction as well. Rotating the cross sample by an angle of 90° allows one to achieve this configuration, and the result is displayed in Figure 13b (red curve). Based on the comparison of the charge generated on the central sensor along two directions (red and blue curves), several relevant points are pointed out, as shown below:

- The orientation of Sensor 3 does affect the charge measure, which manifests in a discrepancy between the radial and axial direction.
- The radial direction of Sensor 3 (equivalent to its width axis) leads to a higher output signal than the axial direction (equivalent to its length axis), because of significant strain variation occurring at the cross’s center.
- The mechanical strain strongly affects the determination of the sensor sensitivity, which must be estimated for the whole area covered by the electrode.
- In reality, the deformation on the bearing might not always be uniform; the shape and orientation of the electrode should be carefully considered.

Note that the output electrical charge of the other side sensors (i.e., Electrodes 1, 2, 8, and 9) in the radial direction is assumed to be negligible as no load is applied on their substrate. This behavior is also confirmed by the numerical simulation described in Figure 6a. Thus, the curve of these sensors was not displayed in Figure 13b. Thanks to the symmetry of the cross shape, the responses of Electrodes 1, 2, 8, and 9 in the axial direction would be similar to the ones of Electrodes 4, 5, 6, and 7 in the radial direction. To sum up, side sensors can be used to monitor unidirectional load, and their output signal is the same for the cross substrate as well as the beam substrate [37]. The central sensor gives out a response in both axial and radial loads, so it could be used as a bidirectional sensing device. From the material point of view, all of these sensors have the same physical characteristics, but depending on how they are implemented with respect to the host structure, they could induce different output behaviors. Last but not least, understanding the sensor’s characteristics is not sufficient; enhancing its design and implementation is of primary importance to fully achieve the sensor’s potential.

**Figure 14:**
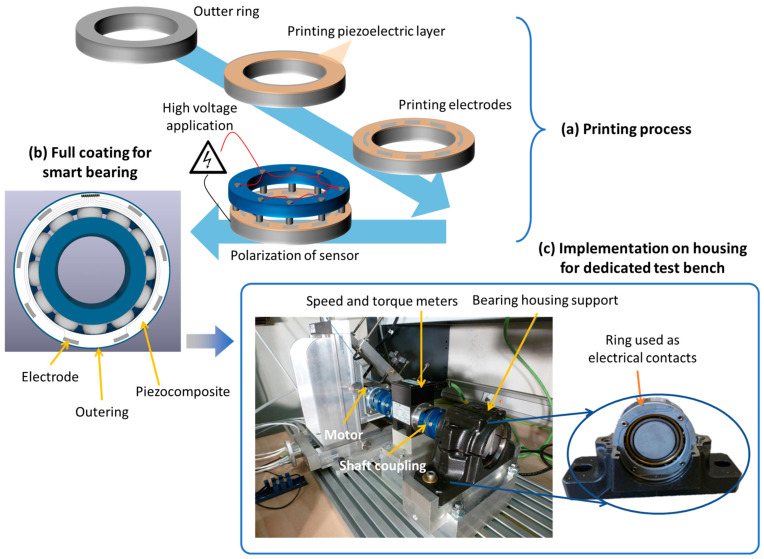
Future developments in smart sensor coating for condition monitoring of applied load: (**a**) printing process, (**b**) full design of smart bearings; and (**c**) implementation in housing for dedicated test bench.

Figure 14 illustrates a future perspective of smart sensor coating used for the condition monitoring of bearings. The printing process of the piezoelectric ink on the outer ring (Figure 14a) is revealed to be similar to what has occurred on the steel substrate (cf. Section 4.2.1). Bearings could be instrumented with a thin-film coating sectorized by eight electrodes with dimensions of L×W=2×8 mm^2^ and 10 µm thickness (Figure 14b). Regarding the important curvature of bearings, the area of each electrode is designed to be small enough to be considered as a flat surface, whose configuration is close to the tested substrate. As a matter of fact, it can be supposed that the force applied on each electrode is almost constant, leading to homogenous deformation induced in each sensing element. This specific design makes it possible to accurately determine the local force exerted on the rolling balls in axial and radial directions. Monitoring charge measurements of all sectorized piezoelectric sensors allow one to determine a complete load mapping of the whole bearing system. Due to the specified geometry of the outer ring, rectangular electrodes were preferred instead of circle ones. The electrical connector together with conductive tracks stacked on a dielectric layer were also included to facilitate measurements as well as to achieve full smart coating with a minimization of any undesired effects. Future development will focus on the implementation of the developed active sensor bearings on a dedicated test bench, together with the assessment of the design performance in a real condition (Figure 14c). In this preliminary test, a static load of 10 kN was applied to the bearing by means of a hydraulic press, in the axial direction only. The inner ring of the bearing was driven via a synchronous electric motor (AKM33H, Kollmorgen) with a rotational speed of 2000 rpm. Two shaft couplings were employed to join the rotational motor, torquemeter (TM series, Magtrol, Switzerland), and the bearing equipment. Different sizes of housing systems were used to fit with several dimensions of bearings. Typically, the bearings used in the laboratory have 15 mm of thickness, and 110 mm and 45 mm of outer ring and inner ring diameters, respectively. The real bearing used in a motor of aircraft is usually bigger.

## 5. Conclusions

This study reported on the development of multilayered sensor coatings using the screen printing technique, which relies on standard design rules of the electronic sensor network. To avoid parasitic capacitances of the printed sensor circuit, the conductive tracks (CTs) were deposited on a dielectric layer while the electrodes were coated on the piezocomposite layer (made of BaTiO_3_ ferroelectric fillers randomly dispersed within Polyurethane Acrylate (PUA) thermoplastic). For a better achievement of reliable measurements, other geometric constraints of CTs were also investigated. A measurement setup was involved, leading to comprehensive electromechanical characterizations of piezoelectric thin-film structures used for the condition monitoring (CM) of bearings. The bearing was assimilated to a cross-shaped steel substrate, which was instrumented by customized piezocomposite sensors capable of delivering linear electrical signals in response to an application of bidirectional load (axial and radial). The experimental setup, including two four-point bending (4PB) sample holders, allowed us to drive the bidirectional strain to the sensor, which in turn implied an output signal by measuring the induced charges. Regarding the strain distribution in the sensing layer computed by a finite element model (FEM), it has been revealed that the center of the cross substrate resulted in a significant drop as opposed to the two sides. This effect can be explained by the fact that the input load was somehow distributed in both axial and radial directions. Consequently, a piezoelectric sensor implemented on the intersecting area of the cross sample is capable of detecting load in both directions. As the strain induced on that area was not homogenous, the orientation and shape of the electrode coated on it could affect the sensor output signal, to some extent. Finally, there are several parameters relating to the structural design of smart sensor coatings that might have an impact on sensing performance. Understanding the ultimate role of each parameter is of crucial importance to achieve reliable condition monitoring of bearings.

Last but not least, the investigation explored in this work is proof of an innovative concept in multidirectional load-sensing bearings, which will be conducted in a real-world operation to validate its reliability for the aeronautic field. To boost the piezoelectric sensitivity, an improvement in the process via dielectrophoresis structuration of the material is envisaged in future work. Other alternatives intend to increase the filler’s concentration with the use of highly anisotropic fillers (e.g., rod or wire shape) to enhance the printed coating performance.

## Figures and Tables

**Table 1:** Parameters of the analytical model.

Material	Properties	Symbol
Steel substrate		
Density	*ρ*	7860 kg/m^3^
Poisson’s Ratio	*v*	0.33
Young’s modulus	*Y*	210 GPa
Width	*w*	20 mm
Height	*h*	1.5 mm
Length	*l*	185 mm
Piezoelectric composite		
Density	*ρ′*	3000 kg/m^3^
Poisson’s Ratio	*v′*	0.37
Young’s modulus	*Y′*	7 GPa
Width	*w′*	20 mm
Height	*h′*	0.2 mm
Length	*l′*	160 mm
Relative permittivity	*ɛ′*	10.5
Piezoelectric constants	*e*_31_ *= e*_33_ *e*_15_	0.016 C/m^2^ 0 C/m^2^

**Table 2:** Mechanical behavior of the cross substrate under three different configurations of the 4PB test.

General observations for all configurations	The induced strain is drastically dropped at the center (defined as intersecting area of the cross sample), as a distribution of load in both directions makes the stress decrease, and so does the strain. At the two sides (out of the intersection), the strain is higher as load contributes to one direction only. The strain manifests a symmetric behavior with respect to the center, which agrees with the symmetric property of the 4PB method and the cross shape. $\mathrm{Three}\;\mathrm{configurations}\;\mathrm{make}\;\mathrm{both}\;S_{x}$$\;\mathrm{and}\;S_{z}$$\;\mathrm{vary},\;\mathrm{but}\;\mathrm{the}\;\mathrm{total}\;\mathrm{strain}\;(S_{x}+S_{z}$) is unchanged and only depends on the load level. For a given load, the total strain of the 2D cross shape is similar to that of the 1D beam shape [37].
Unidirectional load applied along *x*-axis (Figure 6b)	$\mathrm{Strain}\;\mathrm{along}\;x\text{-}\mathrm{axis}\;(S_{x})\;$ attains a plateau at the two sides, which somehow corelates to the constant behavior of the beam substrate within the two points applied by the inner loads [37]. $\mathrm{As}\;\mathrm{the}\;\mathrm{intersecting}\;\mathrm{area}\;\mathrm{is}\;\mathrm{stressed}\;\mathrm{in}\;\mathrm{both}\;\mathrm{directions},\;\mathrm{the}\;\mathrm{strain}\;\mathrm{along}\;\mathrm{the}\;z\text{-}\mathrm{axis}\;(S_{z})\;$$\mathrm{is}\;\mathrm{also}\;\mathrm{varied}\;\mathrm{at}\;\mathrm{the}\;\mathrm{center},\;\mathrm{but}\;\mathrm{with}\;\mathrm{much}\;\mathrm{smaller}\;\mathrm{amplitude}\;\mathrm{and}\;\mathrm{inversed}\;\mathrm{sign}\;(S_{z}>0\;\mathrm{while}\;S_{x}>0)$. $S_{z}$ is negligible at the two sides, as they are out of the intersection that is subjected to free load.
Symmetric bidirectional load (Figure 7b)	$\mathrm{As}\;\mathrm{expected},\;S_{x}$$\;\mathrm{and}\;S_{z}$ are perfectly identical. $\mathrm{At}\;\mathrm{the}\;\mathrm{intersecting}\;\mathrm{area},\;\mathrm{stress}\;\mathrm{is}\;\mathrm{equally}\;\mathrm{distributed}\;\mathrm{along}\;x\text{-}\;\mathrm{and}\;z\text{-}\mathrm{axis},\;\mathrm{leading}\;\mathrm{to}\;\mathrm{smaller}\;S_{x}$ compared to the unidirectional case. Maximum stress is found at the area applied by load. $\mathrm{The}\;\mathrm{distance}\;\mathrm{parameters}\;(L_{x},L_{z}$$,D_{x},D_{z})$$\;\mathrm{strongly}\;\mathrm{affect}\;\mathrm{the}\;\mathrm{allure}\;\mathrm{of}\;\mathrm{the}\;\mathrm{stress}/\mathrm{strain}:\;L_{x}\;\mathrm{and}\;L_{z}$$\;\mathrm{define}\;\mathrm{the}\;\mathrm{width}\;\mathrm{of}\;\mathrm{the}\;\mathrm{maximum}\;\mathrm{peaks}\;\mathrm{while}\;D_{x}\;\mathrm{and}\;D_{z}$ delimit the length of the zone manifested by the deformation (meaning that out of this zone no strain is induced).
Asymmetric bidirectional load (Figure 8a–c)	The following are similar to the last three observations as in the case of “symmetric bidirectional load”: $S_{x}$$\;\mathrm{and}\;S_{z}$ are not the same. The tuning is more flexible, which is based on four distance parameters instead of two (Figure 8c). Modification of parameter in one direction could lead to change in the other direction: see comparison between Figure 7b and Figure 8a (or Figure 8b$)\;\mathrm{where}\;\mathrm{only}\;\mathrm{one}\;\mathrm{parameter}\;\mathrm{in}\;\mathrm{the}\;z\text{-}\mathrm{axis}\;(L_{z}$$\;\mathrm{or}\;D_{z})\;$$\mathrm{is}\;\mathrm{modified}.\;\mathrm{Not}\;\mathrm{only}\;\mathrm{is}\;\mathrm{the}\;\mathrm{allure}\;\mathrm{of}\;\mathrm{the}\;\mathrm{strain}\;\mathrm{in}\;\mathrm{that}\;\mathrm{direction}\;(S_{z})$$\;\mathrm{changed},\;\mathrm{but}\;\mathrm{the}\;\mathrm{one}\;\mathrm{of}\;S_{x}$ is too. For a given value of *D*, a higher value of *L* gives rise to a larger constant plateau on the side area, but a smaller peak (Figure 8a). For a given value of *L*, a higher value of *D* leads to a decrease in the peak level and in the plateau width (Figure 8b).

## Data Availability

Not applicable.
